# Effects of ACE Inhibitors on Insulin Resistance and Lipid Profile in Children with Metabolic Syndrome

**DOI:** 10.4274/Jcrpe.1020

**Published:** 2013-09-18

**Authors:** Eda Çelebi Bitkin, Mehmet Boyraz, Necati Taşkın, Arzu Akçay, Korkut Ulucan, Mehmet Bedir Akyol, Teoman Akçay

**Affiliations:** 1 Van Regional Education and Research Hospital, Division of Pediatrics, Van, Turkey; 2 Fatih University, Faculty of Medicine, Department of Pediatric Endocrinology, Ankara, Turkey; 3 Kanuni Sultan Süleyman Education and Research Hospital, Division of Pediatrics, İstanbul, Turkey; 4 Kanuni Sultan Süleyman Education and Research Hospital, Division of Pediatric Hematology and Oncology, İstanbul, Turkey; 5 Üsküdar University, Faculty of Engineering and Natural Sciences, Department of Molecular Biology and Genetics, İstanbul, Turkey; 6 Dr. Sadi Konuk Education and Research Hospital, Division of Pediatric Cardiology, İstanbul, Turkey; 7 Dr. Sadi Konuk Education and Research Hospital, Division of Pediatric Endocrinology, İstanbul/Turkey

**Keywords:** metabolic syndrome, ACE inhibitor, insulin resistance

## Abstract

**Objective:** The aim of this study was to evaluate the effects of using ACE inhibitors on insulin resistance, glucose metabolism, body fat composition, and lipid profile in children over 10 years of age with obesity-associated metabolic syndrome (MS).

**Methods:** A total of 53 children with MS, who had been followed for at least one year were included in the study. The sample was divided into two groups: Group 1-30 obese children (13 female, 17 male) who were not using an ACE inhibitor and Group 2-23 obese children (13 female, 10 male) who were using an ACE inhibitor. Anthropometric and laboratory dataobtained at baseline and at the 3rd, 6th, and 12th months of follow-up were compared in the two groups.

**Results:** Comparison of the data in the two groups at 3rd, 6th, and 12th months revealed no statistically significant differences in terms of weight standard deviation score (SDS), body mass index SDS, weight for height percentile, body fat percentage, and very low-density lipoprotein (VLDL)values. However, there were statistically significant differences in mean glucose and insulin levels, homeostasis model assessment for insulin resistance, LDL and high-density lipoprotein values, and highly significant differences in mean triglyceride values.

**Conclusions:** The positive effects of ACE inhibitor drugs, particularly on hypertriglyceridemia and insulin resistance, might bring them forth as first-line drugs in the treatment of obese and hypertensive children. Randomized, controlled, double-blind, and long-term studies are needed for a definitive conclusion.

**Conflict of interest:**None declared.

## INTRODUCTION

The worldwide prevalence of childhood obesity has increased greatly over the past 3 decades, and this obesity epidemic is believed to lead to an increasing occurrence of some disorders [e.g. type 2 diabetes mellitus (T2DM)] in children (1,2).

Some individuals are genetically predisposed to insulin resistance. In these individuals, factors such as irregular lifestyles, physical inactivity, unbalanced, and excessive nutrition trigger the development of insulin resistance, a state which ultimately leads to development of the metabolic syndrome (MS) (3). Components characteristic of MS include abdominal obesity, atherogenic dyslipidemia, elevated blood pressure, insulin resistance/glucose intolerance, and prothrombotic and proinflammatory states. Antihypertensive drugs have varying effects on metabolic factors and insulin resistance. While beta blockers and diuretics have known negative effects, calcium channel blockers exhibit neutral effects, and ACE (angiotensin-converting enzyme) inhibitors and angiotensin receptor blockers (ARBs) exhibit neutral or positive effects.

Obesity plays the most important role in the pathophysiology of the MS, a condition which is accompanied by hyperinsulinism/insulin resistance, hypertension, and hyperlipidemia. Recent studies with children and adolescents have shown that the atherosclerotic process begins at an early age and that it is associated with obesity and other components of the MS (4).

The prevalence of MS varies depending on the criteria set forth for the syndrome, and also on the weight and age group of the subjects. Cook et al (5) reported a 4.2% prevalence of MS among children between the ages of 12 and 19 years according to the National Health and Nutrition Examination Survey (NHANES III) data. Studies also indicate that the prevalence of MS is higher in overweight (above 85th percentile for age and sex) and obese (95th percentile for age and sex) children (5,6,7).

There are a number of studies showing a relationship between ACE inhibitors and carbohydrate and lipid metabolism; however, some of the results remain controversial (8,9). The purpose of the current study was to investigate the effects of the use of ACE inhibitors on lipid profile, insulin resistance, and in turn, on development of MS in obese pediatric patients with MS and essential hypertension.

## METHODS

A total of 53 hypertensive or normotensive children with MS who had been followed up at Sisli Etfal Training and Research Hospital Pediatric Endocrinology outpatient clinic for at least one year were included in the study and were evaluated retrospectively. Thirty of these patients (Group 1) were not using and 23 (Group 2) were using ACE inhibitors. Group 1 consisted of 13 female (43.3%) and 17 male (56.7%) children with a mean age of 13.85±1.67 years (distribution: 11.40 - 17.20 years), and Group 2 consisted of 13 female (56.5%) and 10 (43.5%) male children with a mean age of 14.21±1.66 years (distribution: 11.50-17.70 years). The children in Group 2 had been followed up for essential hypertension and were started on treatment with ACE inhibitors (2 x 5mg Enalapril) due to the inability to control their hypertension with exercise and diet.

Children under the age of 10 years, those with MS who were on medication for T2DM, those with pathological findings in thyroid function tests, with additional chronic diseases, and patients with secondary hypertension associated with any other reason were not included in the study.

The criteria set for a diagnosis of MS in children in the current study were (7):

• Body mass index (BMI) (according to age and sex): z-score≥ 2

• Hypertension: diastolic and/or systolic blood pressure>95th percentile

• Triglyceride level : >95th percentile

• High-density lipoprotein (HDL) cholesterol level: <5thpercentile

• Fasting glucose level: ≥ 110mg/dL

Patients who met at least 3 out of the 5 criteria listed above were accepted to have MS.

After obtaining their detailed medical histories, a physical examination including blood pressure measurements, weight and height measurements was performed in all patients. The standard deviation scores (SDS) for body mass index were calculated and percentile values for weight were estimated. Bioimpedance measurements were performed for body fat analysis while the patients were in a fasting state. Fasting glucose and insulin levels, homeostasis model assessment for insulin resistance (HOMA-IR) indices [fasting insulin (mU/L) x fasting glucose (mg/dL) / 405], lipid profiles, and thyroid, liver and kidney functions were also monitored. Adrenocorticotropic hormone and cortisol level measurements were requested only in cases with suspicion for Cushing’s syndrome.

Oral glucose tolerance test was performed in children with fasting blood glucose levels of 100-125 mg/dL, along with hemoglobin A1c (HbA1c) tests. Secondary causes of hypertension, such as renal and cardiac pathologies, were tried to be eliminated in children with blood pressures values above 140/90 mmHg (or >95th percentile according to age). Antihypertensive treatment was not immediately started in patients with hypertension caused by undetermined secondary causes. Instead, these patients were advised to diet and exercise regularly while restricting salt intake. Patients who still had high blood pressure after following these recommendations were started on ACE inhibitors.

Instead of providing a strict diet regimen for the treatment of obesity, the children and their families were informed about healthy nutrition, including the restriction of candies and fast or junk food, and were encouraged to increase physical activity.

Patient follow-ups were planned with intervals of three months. Laboratory and USG tests were repeated depending on the patients’ conditions at follow-up visits.

The findings at the initial (0), 3rd, 6th, and 12th month follow-up visits were compared in terms of sex, age, weight SDS, BMI SDS, body fat percentage, weight for height percentile, fasting insulin and glucose levels, HOMA-IR indices, and lipid profiles.

**Statistical Analysis**

The statistical analyses were performed using the Statistical Package for the Social Sciences (SPSS; SPSS Inc, Chicago, Il, USA) version 13.0. Descriptive statistics were presented as mean ± standard deviation (SD) values. For the analyses of the groups, Student’s t-test (independent sample t-test) was used for homogeneous distribution, and Mann-Whitney U-test was used otherwise. Pearson’s correlation analysis was used for correlation analyses with “r” as the coefficient. Results were evaluated with a 95% confidence interval and a significance level of 0.05 (p < 0.05). For the evaluation of the repeated tests within each group, analysis of variance (ANOVA) was used.

## RESULTS

There were no significant differences between the groups in terms of initial parameters, which were sex, age, weight SDS, BMI SDS, body fat percentage, weight for height percentile, fasting insulin and glucose levels, HOMA-IR indices, and lipid profiles (Tables 1 and 2).

Anthropometric comparison of the groups at 3rd, 6th, and 12th months revealed no statistically significant differences in terms of weight SDS, BMI SDS, and weight for height percentile (Table 3). However, evaluation of the groups for mean laboratory values throughout the follow-up period revealed statistically significant differences in terms of mean glucose and insulin levels, HOMA-IR, low-density lipoprotein (LDL) and HDL values, and highly significant differences in mean triglyceride values. Differences between mean very LDL (VLDL) values were not of statistical significance (Table 4).

## DISCUSSION

The aim of the current study was to evaluate the effects of using ACE inhibitors on MS components. To our knowledge, there were no previous publications investigating the effects of the use of ACE inhibitors or ARBs on glucose metabolism in children at the time of the present study. The relationship between ACE inhibitors and glucose metabolism was first noticed in case reports showing that ACE inhibitors could cause hypoglycemic events in diabetic adult patients using insulin (10,11). These findings were later reinforced with animal and clinical studies which revealed that these agents could prevent progression to diabetes.

The case reports regarding the use of ACE inhibitors in diabetic patients were followed by several case-control studies which documented the relationship between ACE inhibitior and hypoglycemia (8,9,12). In a four-month study comparing the use of captopril and hydrochlorothiazide, the increase in insulin sensitivity was determined to be significantly higher with captopril than with hydrochlorothiazide (13). The decrease in glucose levels being associated with a decrease in insulin levels, and in turn, with a decrease in HOMA-IR indices, supports the findings of previous studies which reported that the decrease in glucose levels with ACE inhibitior was related to the increase in the insulin pathway.

Insulin resistance and visceral fat accumulation are the main characteristics of the MS. Insulin resistance is detected in nearly 50% of hypertensive patients, and as a result of insulin resistance, hyperinsulinemia contributes to the elevation of blood pressure by promoting sympathetic nervous system and renin-angiotensin activities (14).

Some studies have shown the effectiveness of renin-angiotensin blockade in weight loss. In an in vivo study, telmisartan was shown to be effective in preventing weight gain and increasing insulin sensitivity in rats made obese through diet (15). Telmisartan was also reported to have prevented weight gain, reduced accumulation of visceral fat, shrunk adipose cells, and decreased hepatic triglyceride contents in Sprague Dawley rats which had initially gained weight through high-fructose loads (16). In the current study, although there were statistically significant differences between the two groups in terms of increased insulin sensitivity and decreased fasting glucose levels, the difference between the groups was insignificant in terms of, and despite, the higher decrease in body fat percentage and higher ratio of weight loss observed in the ACE inhibitor group. The short follow-up period might partially explain this insignificance. We believe that the decrease in insulin sensitivity can be continuous with the use of ACE inhibitors, and that its long-term use can lead to a significant difference in BMI and body fat percentage. First and foremost, the above-mentioned studies are rat studies conducted under experimental conditions in laboratory settings. Although these studies are invaluable, it is impossible to fully apply their findings to clinical situations since patients, followed in clinical settings, are a heterogeneous population in terms of their lifestyles, dietary habits, and their treatment regimens. It is impossible for humans and rats to be fully congruent in the metabolic sense.

In a meta-analysis of 18 studies evaluating thousands of patients in total, it was concluded that ACE inhibitors decelerate progression to diabetes (17). In the present study, the increase in insulin sensitivity observed in the group treated with ACE inhibitors supports the positive effects of ACE inhibitior on glucose metabolism.

Recently, certain ARBs have been determined to exert their effects on insulin resistance through a mechanism - independent of this effect - other than RAS inhibitior; this mechanism is the partial peroxisome proliferator-activated receptor (PPAR) gamma activation. It was shown that, among ARBs, the PPAR-gamma stimulation was present only in telmisartan at therapeutic concentrations (18). In two separate studies on this matter by Derosa et al (19), conducted on hypertensive type 2 diabetic patients, telmisartan (40 mg/day) was compared with eprosartan (600 mg/day) in one, and with nifedipine gastrointestinal therapeutic system (GITS; 20 mg/day) in the other. At the end of the 12-month treatment period, decreases in total and LDL cholesterol levels were observed with telmisartan in both studies, while no significant effects on glucose metabolism were determined (19). In contrast, Honjo et al (20) compared the effects of telmisartan (20-40 mg/day) and candesartan(8 mg/day) on HbA1c in a small-scale study on 38 Japanese patients with T2DM and determined a significant decrease in HbA1c levels in the telmisartan group. In a study by Miura et al (21), 18 patients with T2DM and hypertension who were being treated with sulphonylurea and previously receiving candesartan (8 mg/day) or valsartan (80 mg/day) for at least 6 months were switched to telmisartan (40 mg/day), which resulted in a significant decrease in fasting insulin and triglyceride levels at the end of the 12-month follow-up period. However, Miura et al (21) also observed statistically insignificant mild decreases in fasting plasma glucose, HbA1c, and total cholesterol levels.

In a placebo-controlled, randomized, prospective study by Nagel et al (22), hypertensive patients with insulin resistance were treated with 40 mg/day telmisartan for 12 weeks. At the end of the treatment period, a significant decrease in insulin resistance was determined using intravenous glucose tolerance tests, but no significant changes were observed in plasma lipids (22). In a case study by Pershadsingh and Kurtz (23), 8 weeks after starting a 52-year-old patient with MS on telmisartan (80 mg/day), glucose and insulin levels returned to normal levels. However, when the patient was switched to 160 mg/day valsartan as antihypertensive treatment, insulin and glucose levels were observed to re-elevate, which, again, returned back to normal once the patients were re-switched back to telmisartan (23). The conclusions of all the above-mentioned studies included positive effects of telmisartan on insulin resistance and diabetes progression.

The effects of antihypertensive agents on MS components are variable. Diuretics and beta blockers are known to have positive effects in the prevention of diabetes progression (24). In contrast, improvement in glucose metabolism and increase in insulin sensitivity in non-diabetic patients treated with antihypertensive drugs containing ACE inhibitors or ARBs were reported to be much better compared to patients treated with a beta blocker and a diuretic regimen (25). Moreover, in large-scale studies, ACE inhibitors and ARBs have been shown to delay the progression of new-onset diabetes (25,26).

More studies are needed in order to recommend the use of ACE inhibitors and ARBs as first-line drugs in the treatment of MS. Clinical studies that will provide further evidence on the metabolic benefits of these drugs are still ongoing in adults, but to our knowledge, there are currently no ongoing studies on the metabolic effects of these drugs in children. To our knowledge, the present study was the first one to present the effects of ACE inhibitors on the components of MS in childhood. However, one limitation of the current study was that the data were collected retrospectively. Another limitation was that no obese children using an antihypertensive drug other than an ACE inhibitor were included in the study. Furthermore, the study did not include any data on the patients’ daily calorie intakes, nutritional habits and exercise status. Another limitation of the present study was the smallness of the sample, which was not adequate for a power statistical analysis, and sorting of the groups by gender and Tanner staging was not attempted. This was the major limitation of the present study. Nevertheless, we were able to show the benefits of using ACE inhibitors on the most important criteria of MS, namely, hypertriglyceridemia and insulin resistance. Randomized, controlled, double-blind, and long-term studies are needed for a definitive conclusion on the use of ACE inhibitors as first-line drugs in the treatment of obese and hypertensive children. 

## Figures and Tables

**Table 2:**
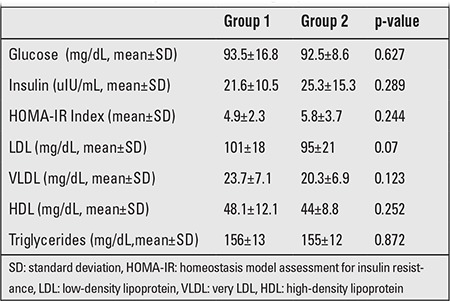
Initial laboratory values in the two groups

**Tablo 1:**
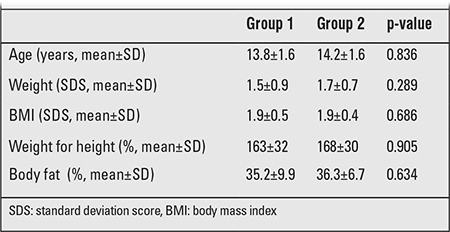
Initial anthropometric measurements in the two groups

**Tablo 3:**
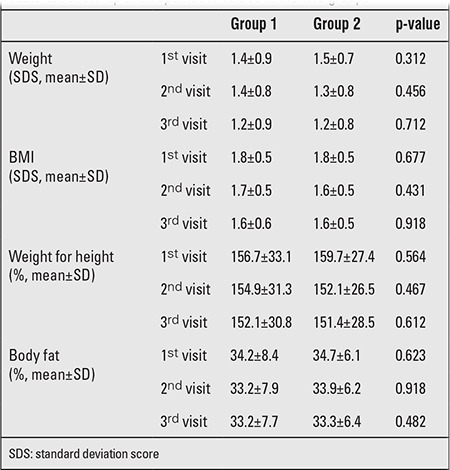
Follow-up anthropometric values in the two groups

**Tablo 4:**
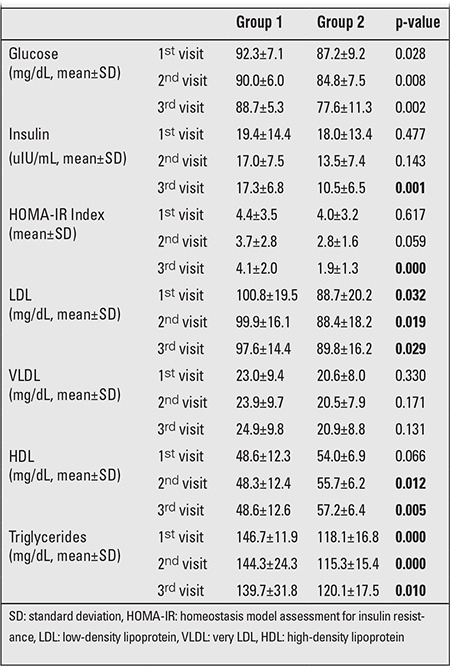
Follow-up laboratory values in the two groups
